# Effect of ceramic and resin cement type on color stability and translucency of ceramic laminate veneers for diastema closure: an in vitro study

**DOI:** 10.1038/s41598-022-26581-5

**Published:** 2022-12-21

**Authors:** Eman Adel Elkhishen, Walid Al-Zordk, Mahy Hassouna, Ahmed Elsherbini, Amal Abdelsamad Sakrana

**Affiliations:** 1 Department of Fixed Prosthodontics, Mansoura University, Faculty of Dentistry, Mansoura, Egypt; 2 Department of Fixed Prosthodontics, Horus University, Faculty of Dentistry, New Damietta, Egypt; 3 Department of Oral-Maxillofacial Surgery, Dentistry and Orthodontics, The University of Tokyo, Tokyo, Japan

**Keywords:** Health care, Materials science

## Abstract

To investigate the effect of resin cements on the color stability and translucency of ceramic laminate veneers used for diastema closure. Sixty resin abutments were prepared for ceramic laminate veneers and divided into six groups according to the ceramic type (lithium disilicate, zirconia-reinforced lithium silicate, and translucent zirconia) and the cement type (*Variolink Esthetic LC and RelyX Veneer*). Color coordinates and translucency were analyzed after cementation and after soaking in the coffee solution. Differences in color and translucency were estimated, and results were statistically assessed (α = 0.05). Ceramic materials showed a significant impact on color changes after soaking in coffee within *Variolink Esthetic* groups. Translucent zirconia showed the highest color change, followed by zirconia reinforced lithium silicate and lithium disilicate. Ceramic materials showed a significant impact among the *RelyX Veneer* groups. A significant interaction in color changes was found between ceramic types and cement types after cementation, and after soaking in coffee was found. All groups showed a clinically acceptable difference in translucency parameters after soaking in coffee. The resin cement affects the color and translucency of ceramic laminate veneers used for diastema closure, and ceramic laminate veneers bonded with *Variolink Esthetic LC* resin cement are more translucent, while ceramic laminate veneers bonded with *RelyX Veneer* resin are more resistant to coffee staining. The lithium disilicate laminate veneer is more resistant to coffee staining than zirconia reinforced lithium silicate and translucent zirconia laminate veneers used for diastema closure.

## Introduction

Ceramic laminate veneers represent a more popular treatment method for many esthetic dental problems, such as diastema closure, due to their high esthetic properties, superior color stability, biocompatibility, documented clinical performance, and good mechanical properties than direct resin composite restoration^1–3^. Diastema, recently considered a malocclusion, has been described as a space equal to or greater than 0.5 mm interdentally and present in most cases between the anterior teeth in the maxilla more than the mandible^4^. Many patients are dissatisfied with the presence of diastema, which negatively attracts attention and spoils the smile^5^. The presence of diastema is an esthetically challenging clinical situation that could be managed by surgery, orthodontic treatment, restorative and fixed prosthodontic approaches, or a combination of the aforesaid^6^. Although the orthodontic approach, as a conservative treatment modality, is recommended in certain cases for diastema closure, it may not be the preferred choice for the patient due to the long treatment time, the appearance of brackets, and the possibility of relapse particularly in midline diastema^1,4^. Direct composite resin can be used to close diastema in a single visit with minimal or no preparation, however it is a sensitive technique and requires a skillful operator^6^. In addition, direct composite laminate veneers may need to be replaced periodically due to shade and texture changes^1^. Regarding the prosthodontic approaches, ceramic crowns provide excellent and long-lasting results but may require extensive tooth preparation^1^. While several treatment options for diastema closure can be presented to the patient, a careful diagnosis is required to formulate the conservative and predictable treatment plan.

Various ceramic systems such as lithium disilicate, lithium disilicate derivatives, and zirconium oxide ceramics have been introduced to the dental market^7–10^. The unique microstructure of lithium disilicate ceramics plays a key role in its mechanical and optical properties^11–14^. Ceramic laminate veneers, if properly bonded, become an integral part of tooth structure and share a portion of the applied load during chewing cycles^15–17^. The resin cement is exposed to dynamic stress and thermal changes, as well as the hydrolytic effect of water and other substances in the mouth^18^. The longevity of ceramic laminate veneers depends on a number of factors, of which optical characteristics are the most important^19,20^. Translucency of a ceramic restoration is influenced by several factors such as the microstructure, thickness, and surface properties of the ceramic material, the illuminant, and the reflectance value of the background. Additionally, the translucency of silicate ceramics is dependent on the heat treatment temperature that induces crystal nucleation and growth process. The thickness of ceramic laminate veneers primarily affects light transmission^13^.

Light cure resin cements with better color stability are recommended to be used for the cementation of ceramic laminate veneers, since the color change of dual cure cements would affect the final esthetic appearance of these restorations^21^. The discoloration of resin cements is influenced by factors, such as filler type, resin matrix, photoinitiator, polymerization, and degree of conversion^22^. Maximum conversion of uncured resin, which depends on the ability of photoinitiators to absorb light during polymerization process, is required since the uncured monomers result in undesirable discoloration of resin cement^23^. Camphorquinone, the most commonly used photoinitiator in light cure resin materials and which requires a co-initiator such as tertiary amine, could cause yellowing of the cured material due to oxidation of amine impurities, affecting the color stability over time^24^. Alternative photoinitiators based on iodonium salts and benzoyl germanium substances that do not require co-initiators were developed and showed low water solubility and significant absorption in visible light, compared to camphorquinone^25–30^.

The color properties and color stability of the ceramic laminate veneers are just as important and critical as their mechanical properties^31,32^. Tooth color analysis is performed to provide a numerical record of the analyzed color and can be described by color systems including Munsell color system and CIELAB color system^33^. The color difference that can be detected by the human eye is perceptibility, but acceptability refers to clinically acceptable color differences^34–36^. The importance of perceptibility and acceptability thresholds stems from their ability to assess the degree of match or mismatch of restorations in color and translucency for the selection and assessment of their clinical performance^37–39^. The final color of a ceramic restoration is affected by the color of resin cement and substrate, if the thickness of the restoration is less than 1.5 mm. The color of substrate, the microstructure of ceramic material, ceramic thickness and resin cement all affect the final esthetic outcome of ceramic laminate veneers^40,41^. Coffee is common in people's daily diet and is recognized as one of the beverages with elevated risk of discoloration^37,42,43^.

Closing a diastema may require more tooth preparation in the interproximal area to allow technician greater freedom in the alteration of the tooth form^41^. Therefore, the purposes of this in vitro study are to investigate; (1) the influence of resin cement with different photoinitiating systems based on benzoyl germanium compounds and iodonium salts on the color and translucency of lithium disilicate, zirconia-reinforced lithium silicate, and translucent zirconia laminate veneers used for diastema closure, and (2) the influence of soaking in coffee solution on the color stability and translucency of ceramic laminate veneers used for diastema closure bonded with resin cements containing different photoinitiators. The first null hypothesis is that there is no statistically significant effect on color and translucency between various ceramic laminate veneering materials cemented with resin cements with different photoinitiators. The second null hypothesis is that there is no effect of soaking in coffee on the color stability and translucency of different ceramic laminate veneers.

## Materials and methods

**Figure 1:**
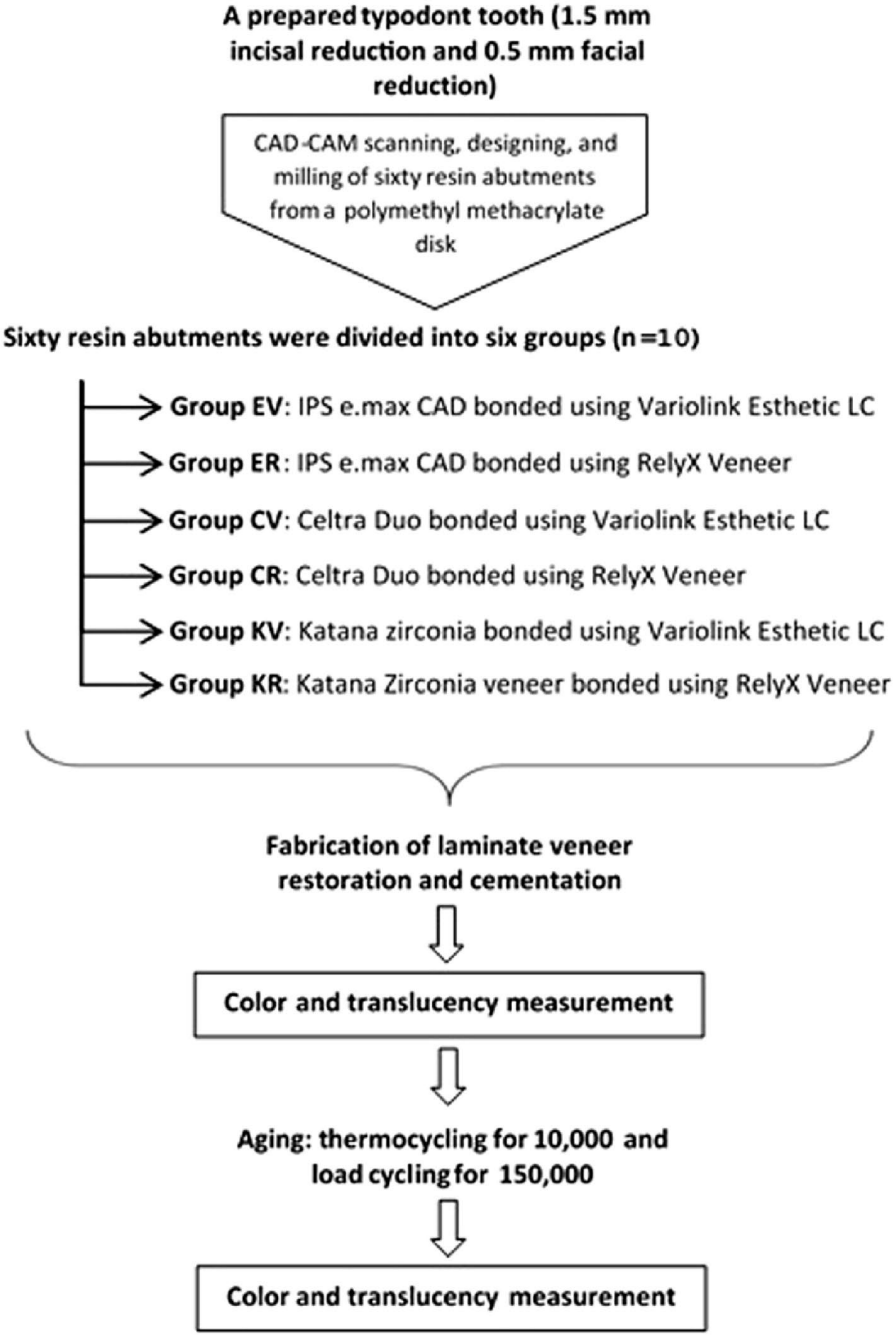
Schematic representation of the main study steps and grouping.

**Figure 2:**
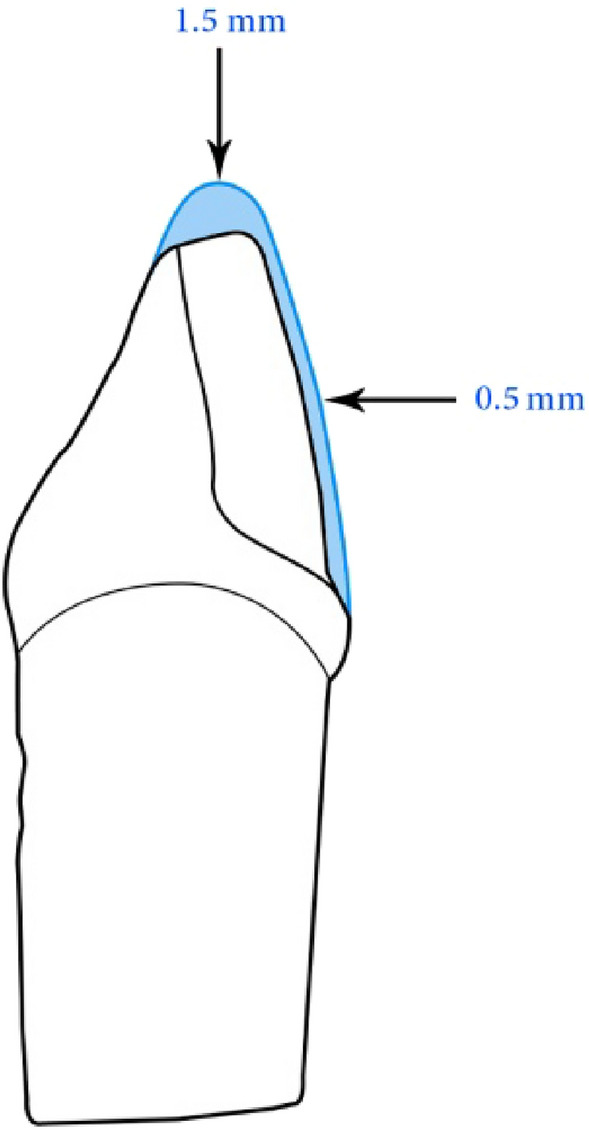
Illustration of the tooth preparation for ceramic laminate veneer with 1.5 mm incisal reduction, 0.5 mm uniform facial reduction extending beyond the proximal contact area, and 0.5 mm chamfer margin.

**Figure 3:**
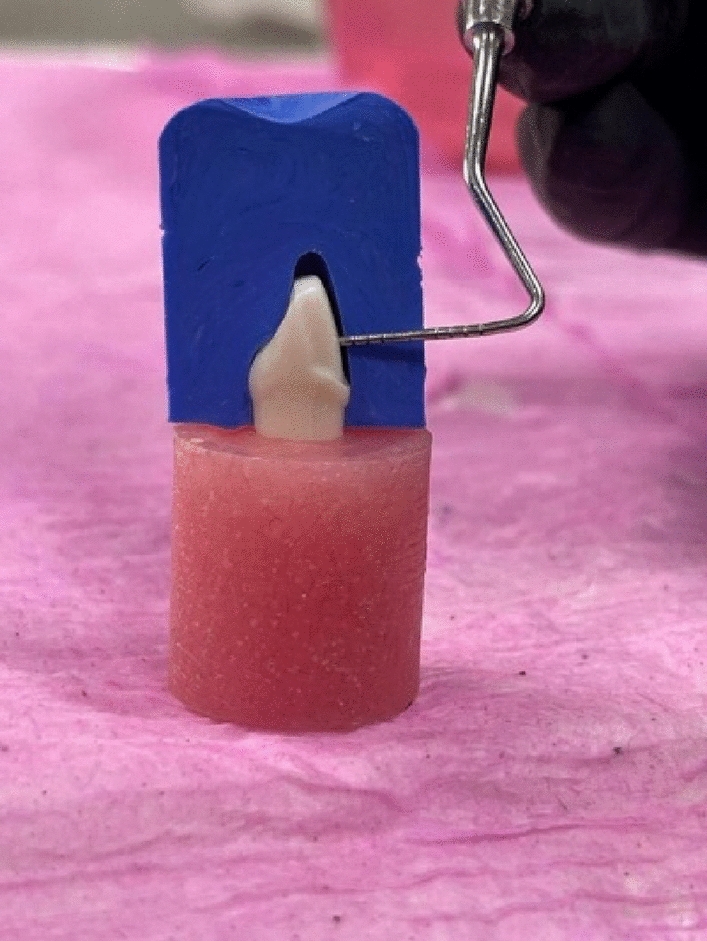
The putty index during checking the tooth preparation of the master abutment for ceramic laminate veneer.

The main steps of the study are presented in Fig. 1. A maxillary left central incisor tooth model (A5A-200, Nissin Dental Products Inc, Kyoto, Japan) was selected as the master abutment, and a pre-preparation putty index was fabricated using vinyl polysiloxane impression material (Imflex Putty, Meta Biomed Co, Chungcheongbuk-do, Korea)*.* The ceramic laminate veneer with butt joint design was prepared by 1.5 mm incisal reduction, facial reduction of 0.5 mm extended beyond the proximal contact area, and 0.5 mm chamfer margin (Fig. 2). The preparation was performed using a specific veneer preparation kit (REF 10.801.002, Microdont, Sao Paulo, Brazil) by a single operator. Finally, the preparation of the master abutment was evaluated using the pre-preparation putty index (Fig. 3).

The prepared tooth model was used to mil sixty resin abutments of the same dimension and color from a polymethyl methacrylate disk (PMMA CAD/CAM Disk-A2 shade, Yamahachi Dental MFG Co., Gamagori, Japan) using CAD-CAM technology. First, the prepared tooth model was scanned using a lab scanner (Freedom UHD Model Scanner, DOF Inc, Seoul, South Korea) and an anti-reflection scan powder (3D Scanning Spray, Bilkim Ltd. Co, İzmir, Turkey) was used. The scanned data was then used to duplicate sixty abutments with the same dimensions using CAD-CAM software (CORiTEC iCAM V5 Smart, Imes-Icore GmbH, Eiterfeld, Germany). Finally, the abutments were milled using the milling machine (CORiTEC 350i Loader pro, Imes-Icore GmbH, Eiterfeld, Germany).

### Study grouping

According to the ceramic materials and cement types (Table 1), the milled abutments were divided into six groups (n = 10) as follows; Group EV: *IPS e.max CAD /A2* (Ivoclar Vivadent, Schaan, Liechtenstein) bonded using *Variolink Esthetic LC /Neutral* (Ivoclar Vivadent, Schaan, Liechtenstein), Group ER: *IPS e.max CAD* bonded using *RelyX Veneer /TR* (3M ESPE, MN, USA), Group CV: *Celtra Duo /A2* (Dentsply Sirona, NC, USA) bonded using *Variolink Esthetic LC*, Group CR: *Celtra Duo* bonded using *RelyX Veneer*, Group KV: *Katana zirconia UTML /A2* (Kuraray Noritake Dental Inc, Tokyo, Japan) bonded using *Variolink Esthetic LC*, and Group KR: *Katana Zirconia* veneer bonded using *RelyX Veneer* (Fig. 1).

**Table 1:** Characteristics of materials used in the study.

Material	Batch number	Manufacturer	Composition (wt%)
**Ceramic material**			
IPS e.max CAD (HT/A2)	Z018J6	Ivoclar Vivadent, Schaan, Liechtenstein	SiO_2_ (57–80%), Li_2_O (11–19%), K_2_O (0–13%), P_2_O_5_ (0–11%), ZrO_2_ (0–8%), ZnO (0–8%), Al_2_O_3_ (0–5%), MgO (0–5%), and pigments (0–8%)
Celtra Duo (HT/A2)	16004697	Dentsply Sirona, NC, USA	SiO_2_ (58%), ZrO_2_ (10%), P_2_O_5_, Al_2_O_3_, Li_2_O, and ZnO
Katana Zirconia UTML (A2)	DZMNQ	Kuraray Noritake Dental Inc, Tokyo, Japan	ZrO_2_ + HfO_2_ (87–92%), Y_2_O_3_ (8–11%), and pigments (0–2%)
Resin cement			
Variolink Esthetic LC (Neutral shade)	Y22403	Ivoclar Vivadent, Schaan, Liechtenstein	Filler (60–58% ytterbium trifluoride and spheroid mixed oxide), monomer mixture of UDMA, Bis-GMA, HEMA, TEGDMA, and GDMA (30–38%), initiator and stabilizer (1–2% Ivocerin, and thiocarbamide hydroperoxide), and pigments (< 1%)
RelyX Veneer (translucent shade)	NC12805	3M ESPE, MN, USA	Filler (55–65% silane treated ceramic and 1–10% silane treated silica), Bis-GMA (10–20%), TEGDMA (10–20%), EDMAB, Diphenyliodonium hexafluorophosphate (< 0.5), Benzotriazole, pigments (< 1)

*PMMA* polymethyl methacrylate, *UDMA* urethane dimethacrylate, *Bis-GMA* bisphenol A-glycidyl methacrylate, *HEMA* hydroxyethyl methacrylate, *TEGDMA* tri ethylene glycol di methacrylate, *GDMA* glycerol dimethacrylate, *EDMAB* ethyl-4-dimethylamino benzoate, *MDP* methacryloyloxydecyl dihydrogen phosphate, *HEMA* hydroxyl methacrylate.

### Fabrication of ceramic laminate veneers

To fabricate ceramic laminate veneers, all abutments were sprayed with anti-reflection scanning powder spray (3D Scanning Spray, Bilkim Ltd. Co, İzmir, Turkey) and then scanned with the CAD-CAM scanner (Identica hybrid, Medit, Seoul, Korea)*.* Each ceramic laminate veneer was designed using CAD-CAM software (Collab 2017, Exocad, Darmstadt, Germany). The pre-preparation scan was used to support the transfer and standardize the design for all restorations. To close a 1 mm diastema from both sides, the proximal thickness of the design was gradually increased from cervical to incisal, with a maximum increase in thickness in the contact area (0.5 mm). Each designed restoration had a cement gap thickness of 50 µm, a uniform facial thickness of 0.5 mm, and an incisal thickness of 1.5 mm. The ceramic laminate veneers were then milled using the milling machine (CORiTEC 350i Loader pro, Imes-Icore GmbH, Eiterfeld, Germany). The milled *IPS e.max CAD* laminate veneers were finished and then subjected to crystallization and glaze firing in the porcelain furnace (Pogramat P500, Ivoclar Vivadent, Schaan, Liechtenstein) according to the recommendations of the manufacturer. The milled *Celtra Duo* veneers were cleaned and then subjected to glaze firing following the instructions of the manufacturer. The milled zirconia laminate veneers were sintered at 1550 °C with a holding time of 2 h in a sintering furnace (Tabeo, Mihm-VOGT, Stutensee, Germany). The zirconia laminate veneers were then glazed (Cerabien ZR Glaze, Kuraray Noritake Dental Inc, Tokyo, Japan) and subjected to glaze firing following the instructions of the manufacturers. Finally, the digital caliper was used to verify the dimensions of each restoration.

### Cementation

The intaglio surfaces of *IPS e.max CAD* and *Celtra Duo* laminate veneers were etched using hydrofluoric acid (Porcelain Etch, Ultra Dent, Utah, USA) for 20 s, rinsed and air dried. A thin coat of universal primer (Monobond N, Ivoclar Vivadent, Schaan, Liechtenstein) was then applied to the etched surfaces with a disposable brush for 60 s and allowed to air dry for 10 s. The intaglio surfaces of zirconia laminate veneers were air-borne particle abraded (Basic eco, Renfert, Hilzingen, Germany) with 50 μm alumina (Al_2_O_3_ Cobra, Renfert, Hilzingen, Germany) at 2.5 bar for 10 s/cm^2^ at a distance of 10 mm and perpendicular to the surface. The prepared surfaces of the abutment were treated with 37% phosphoric acid (N-Etch, Ivoclar Vivadent, Schaan, Liechtenstein) for 45 s, washed and air-dried for 10 s^44^.

For EV, CV, and KV groups, the bonding agent (Tetric N-Bond Universal, Ivoclar Vivadent, Schaan, Liechtenstein) was applied according to the manufacturer's instructions, then cured for 10 s with a light curing device (Bluelex LD-105, Monitex Industrial Co., Ltd, New Taipei, Taiwan) and an average light intensity of 1000 mW/cm^2^. The light intensity of the curing device was measured using a radiometer (Bluephase Meter II, Ivoclar Vivadent, Schaan, Liechtenstein) before each application. During the cementation, the resin cement paste (Variolink Esthetic LC Neutral, Ivoclar Vivadent, Schaan, Liechtenstein) was applied to the pre-treated intaglio surface of the restoration, seated on the abutment, and retained under a static load of 250 g^45^. An initial light-curing (Bluelex LD-105, Monitex Industrial Co., Ltd, New Taipei City, Taiwan) was performed for 2 s, and the excess cement was removed. The margins were covered with a glycerin gel (Liquid Strip Glycerin Gel, Ivoclar Vivadent, Schaan, Liechtenstein)*,* then the final curing was performed for 30 s per each surface. For ER, CR, and KR groups, the bonding agent (Single Bond Universal Adhesive, 3M ESPE, Minnesota, United States) was applied and cured for 10 s. The resin cement paste (RelyX Veneer TR, 3M ESPE, MN, USA) was applied to the pre-treated intaglio surface of the ceramic laminate veneer, then seated on its corresponding abutment and cured as described earlier. After cementation, the margins of each restoration were finished and polished with silicone tips (Kenda Dental Polishers, Vaduz, Liechtenstein). All specimens were stored in distilled water at 37 °C for 24 h after cementation.

### Color and translucency measurement (after cementation)

**Figure 4:**
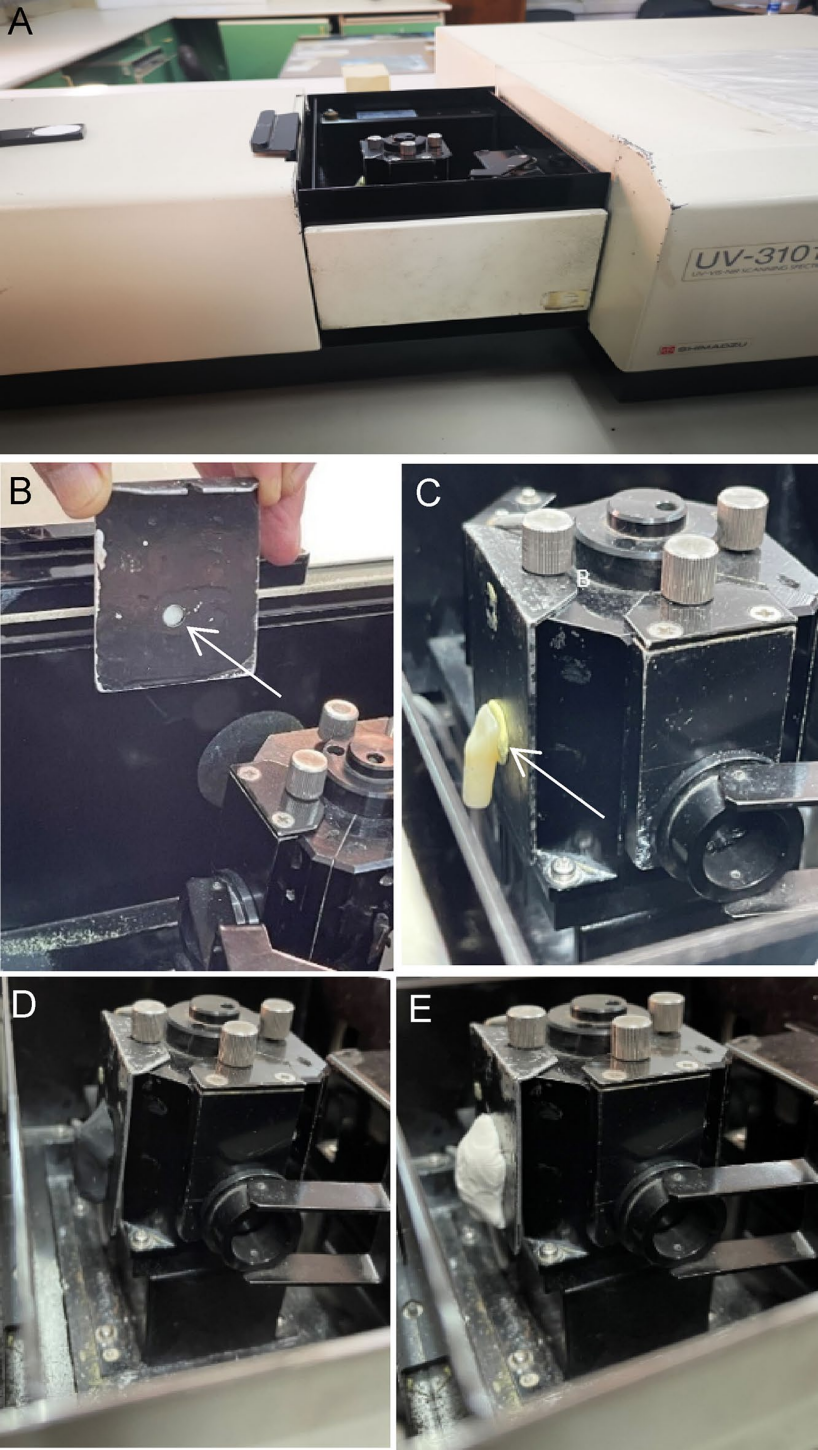
The color measurement. (**A**) The photometer unit of the spectrophotometer device, (**B**) the middle of the facial surface of the specimen fixed on the outer surface of the holder (4-mm diameter), (**C**) the holder with the specimen positioned in its place inside the spectrophotometer, and (**D**) back and white plasticine covering the specimen during measurements.

A reflectance spectrophotometer (UV-Shimadzu 3101 PC, Agilent Technologies Inc, CA, USA) was used to measure and calculate the International Commission on Illumination (CIE) tristimulus color values (Fig. 4). The spectrophotometer was calibrated according to manufacturer's instructions before and after each group color measurement. The CIE D_65_ standard illuminant was selected, as well as a 10° standard observer angle. Color measurements were performed by a single investigator experienced with this device at the middle of the facial surface of the specimen, with a measuring area of 4 mm diameter to reduce edge loss. To secure the specimen in a well-adapted precise position, a self-adhesive pad (Patafix yellow, UHU GmbH Co. KG. Bühl, Germany) was used, along with black plasticine (Jovi, Barcelona, Spain) as backing with the CIE values: L^*^ = 25.87, a^*^ = −1.12, and b^*^ = 0.95. The calculations were carried out according to the CIELAB color system^46^. The CIE coordinates were determined, the measurements were repeated three times for each specimen and the average result was recorded. The CIE coordinates of the specimen were evaluated against black and white settings to obtain the translucency parameter (TP_00_). L^*^ = 25.87, a^*^ = −1.12 and b^*^ = 0.95 are the CIE values for the black setting, while L^*^ = 69.79, a^*^ = 0.03 and b^*^ = 2.70 are the CIE values for the white setting. The following formula was used to calculate the TP_00_ for each specimen^47^:

$$T{P}_{00}= \sqrt{ \left(\frac{{L'}_{B}-{L'}_{w}}{{k}_{L}{S}_{L}}\right)+\left(\frac{{C'}_{B}-{C'}_{w}}{{k}_{C} {S}_{C}}\right)+\left(\frac{{H'}_{B}-{H'}_{w}}{{k}_{H} {S}_{H}}\right)+{R}_{T}\left(\frac{{C'}_{B}-C'w}{{k}_{C} {S}_{C}}\right)+ \left(\frac{{H'}_{B}-{H'}_{w}}{{k}_{H} {S}_{H}}\right)}$$

$L{'}_{B} - L{'}_{W}$ represents the difference in value among black and white settings; $C{'}_{B} - C{'}_{W}$ stands for the difference in chroma in black and white settings; $H{'}_{B} - H{'}_{W}$ represents the difference in hue under black and white settings. The rotation factor and the weighting function are given by R_T_ and S. The parametric factors are indicated by K_L_, K_C_, and K_H_. Each measurement was performed three times, with the average value being recorded. The translucency perceptibility threshold was set at 0.62, while the translucency acceptability threshold was set at 2.62^48^. The contrast ratio was calculated using the following formula:

$$CR=Yb/Yw$$

The CIE tristimulus values of the specimen over black background are represented by Y, while the CIE tristimulus values of the specimen over white background are represented by Yw.

### Aging

The specimens were subjected to 10,000 thermocycled cycles to simulate one-year aging. The dowel time was 20 s in the thermocycling machine between 5 and 55 °C (SD Mechatronic Thermocycler; Germany). The specimens were then subjected to load cycling (Model ACH-09075DC-T, AD-Tech Technology CO, Germany)*.* To mimic one year of masticatory forces in the anterior dentition, the specimens were loaded with 49 N at a rate of 1.6 Hz for 150,000 cycles^49^. A 5.4-mm steel piston was used to deliver the load to the cingulum of the tooth (3 mm below the incisal edge) at a descending speed of 40 mm/s. The specimens were examined for cracks after aging. Then, the specimens were soaked in coffee solution for 18 h after aging^50^. The coffee solution was prepared by combining 15 gm of coffee with 300 mL of water in a filter coffee machine. The specimens were then cleansed by brushing each specimen circumferentially ten times with toothpaste (Sensodyne, GalaxoSmithKline) under running water. Finally, all the specimens were ultrasonically cleaned (Jeken PS-30, Jeken, Guangdong, China) and dried.

### Color and translucency measurement (after aging)

Color and translucency measurements were performed a second time for all specimens as previously described. The color differences following the CIE DE2000 color difference formula (ΔE_00_) were calculated:

$$\Delta {E}_{00}=\sqrt{ \left(\frac{\Delta L}{{k}_{L}{s}_{L}}\right)+\left(\frac{\Delta c}{{k}_{c}{s}_{c} } \right)+\left(\frac{\Delta H}{{k}_{H}{s}_{H} } \right)+{R}_{T}\left(\frac{\Delta c}{{k}_{c}{s}_{c} } \right)+\left(\frac{\Delta H}{{k}_{H}{s}_{H} } \right)}$$

ΔL, ΔC, and ΔH represent lightness, chroma, and hue differences, respectively. R_T_ and S refer to the rotation factor and the weighting function. K_L_, K_C_, and K_H_ represent parametric factors. ΔE_00_ value of 1.30 was considered the perceptibility threshold; while 2.25 was considered the acceptability threshold^51^.

### Statistical analysis

Statistical interpretations were performed utilizing statistical software (SPSS Statistics for Windows v22.0, SPSS Inc, IBM Corp, NY, USA). The Shapiro–Wilk test indicated that the data were normally distributed (*P* < 0.05). The one-way ANOVA test was used to compare more than two independent groups. The interaction of the two independent variables (material type and cement type) and the effect of each variable on the color changes and translucency were assessed using the two-way ANOVA test. The post-hoc Tukey test was also used for multiple comparisons. Significance of the results obtained was judged at *P* < 0.05.

## Results

**Figure 5:**
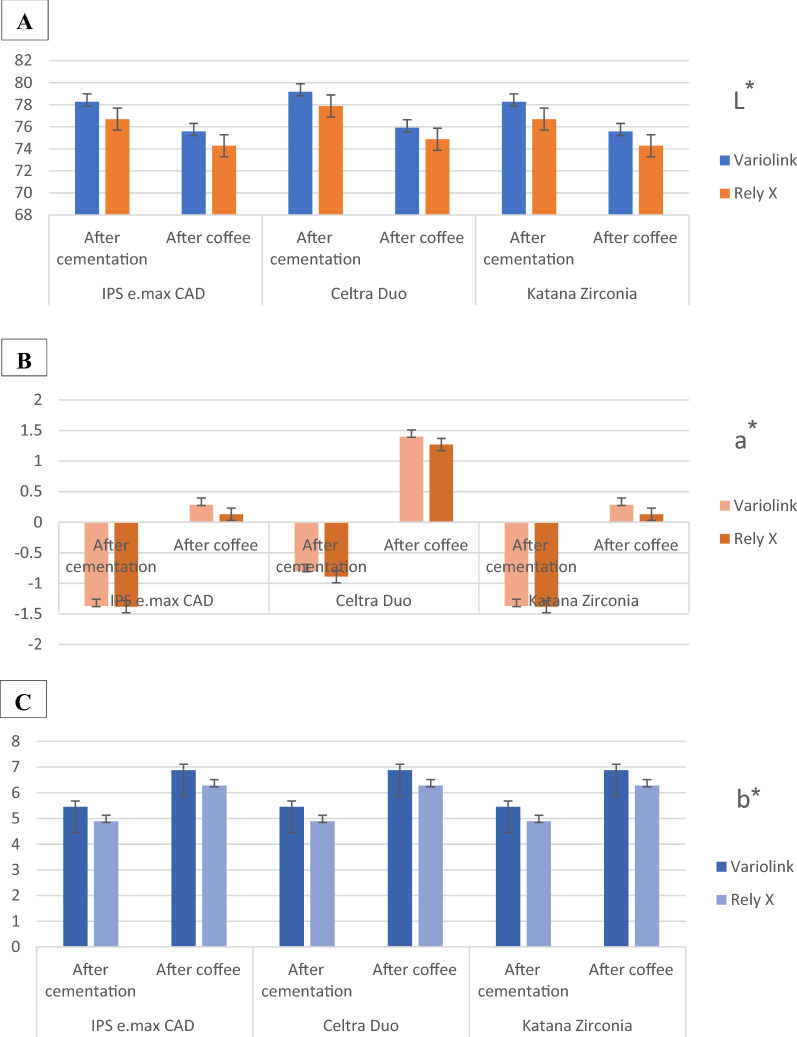
The CIE color coordinates for IPS e.max CAD, Celtra Duo, and Katana zirconia restorations after cementation and after soaking in coffee solution. (**A**) L* (lightness of color), (**B**) a* (red-green axis), and (**C**) b* (yellow-blue axis).

The mean values of the CIE L*, a*, b* color coordinates for *IPS e.max CAD*, *Celtra Duo*, and *Katana zirconia* laminate veneers after cementation and after aging are shown in Fig. 5. The mean values of the color coordinate differences (ΔL*, Δa*, and Δ b*) of the groups examined are presented in Table 2. The two-way ANOVA test (Table 3) showed no interaction was found on ΔL* (*P* = 0.147), but a significant interaction was found between the materials of ceramic laminate veneers and the cement type on Δa* (*P* = 0.015), and Δb* (*P* = 0.001).

**Table 2:** Mean values of differences in lightness (ΔL*), red-green axis (Δa*), and yellow-blue axis (Δb*) of study groups.

Material		Variolink Esthetic	Relay X Veneer	*P*
IPS e.max CAD	ΔL*	−2.68 ± 0.07^A^	–2.41 ± 0.05^a^	˂0.001
	Δa*	1.66 ± 0.06^B^	1.51 ± 0.07^b^	˂0.001
	Δb*	1.43 ± 0.05^C^	1.39 ± 0.05^c^	0.064
Celtra Duo	ΔL*	−3.25 ± 0.11^A^	−3.02 ± 0.08^a^	˂0.001
	Δa*	2.19 ± 0.07^B^	2.16 ± 0.05^b^	0.168
	Δb*	1.94 ± 0.09^C^	1.71 ± 0.07^c^	˂0.001
Katana Zirconia	ΔL*	−3.92 ± 0.09^A^	−3.58 ± 0.09^a^	˂0.001
	Δa*	2.26 ± 0.07^B^	2.11 ± 0.07^b^	˂0.001
	Δb*	2.16 ± 0.07^C^	1.95 ± 0.07^c^	˂0.001

Groups with same uppercase letter in the same column indicate significant difference (*P*˂0.05). Groups with same lowercase letter in the same column indicate significant difference (*P*˂0.05).

**Table 3:** Two-way ANOVA test for impact of ceramic material and resin cement type on ΔL*, Δa*, and Δb*

Source	Dependent variable	Type III sum of squares	df	Mean square	F	P
Corrected model	ΔL*	15.687	5	3.137	412.743	˂0.001
	Δa*	5.046	5	1.009	212.991	0.001
	Δb*	4.773	5	0.955	202.303	0.001
Intercept	ΔL*	593.965	1	593.965	7.8144	˂0.001
	Δa*	235.699	1	235.699	4.9754	0.001
	Δb*	186.702	1	186.702	3.9564	0.001
Ceramic material	ΔL*	14.487	2	7.243	952.885	˂0.001
	Δa*	4.800	2	2.400	506.605	0.001
	Δb*	4.271	2	2.135	452.542	0.001
Resin cement	ΔL*	1.170	1	1.170	153.978	˂0.001
	Δa*	0.202	1	0.202	42.602	0.001
	Δb*	0.400	1	0.400	84.801	0.001
Ceramic material * resin cement	ΔL*	0.030	2	0.015	1.984	0.147
	Δa*	0.043	2	0.022	4.572	0.015
	Δb*	0.102	2	0.051	10.814	0.001
Error	ΔL*	0.410	54	0.008	–	–
	Δa*	0.256	54	0.005	–	–
	Δb*	0.255	54	0.005	–	–

Regarding the color difference, the mean color changes after soaking in the coffee solution were above the acceptability threshold (∆E_00_ ˃ 2.25) for all groups. The two-way ANOVA test detected a significant interaction (*P* = 0.005) between the ceramic material and the cement type. The Post Hoc Tukey test revealed that the lowest color change was related to the lithium disilicate veneers. Within the *Variolink Esthetic* groups, the ceramic material had a significant effect (*P* ˂ 0.001), *Katana zirconia* showed the highest color change (4.49 ± 0.08) followed by *Celtra Duo* (4.12 ± 0.14) and *IPS e.max CAD* (3.25 ± 0.03). The ceramic material also had a significant effect (*P* ˂0.001) in the *RelyX Veneer* groups, where *Katana zirconia* showed the highest color change (4.14 ± 0.07), followed by *Celtra Duo* (3.96 ± 0.11) and *IPS e.max CAD* (3.01 ± 0.07).

The mean values for the translucency parameter (TP_00_) are shown in Table 4. The *IPS e.max CAD* restorations showed the highest mean TP_00_ values within the *Variolink Esthetic* groups after cementation and after coffee staining. However, *Celtra Duo* showed the highest TP_00_ values Within the *RelyX Veneer* groups after cementation, while *IPS e.max CAD* restorations showed the highest value after coffee staining. The two-way ANOVA test discovered a significant interaction between the ceramic material and the cement type after cementation (*P* = 0.040) and after coffee staining (*P* = 0.001).

**Table 4:** Mean translucency parameter (TP_00_) ± standard deviation of tested groups after cementation and after soaking in coffee solution.

Material		Variolink Esthetic	Relay X Veneer	*P*
**After cementation**				
IPS e.max CAD		2.48 ± 0.09^A^	1.95 ± 0.09^a^	˂0.001
Celtra Duo		2.41 ± 0.09^A^	2.02 ± 0.11^a^	˂0.001
Katana Zirconia		1.55 ± 0.08^A^	1.12 ± 0.02^a^	˂0.001
**After soaking in coffee solution**				
IPS e.max CAD		1.86 ± 0.05^B^	1.37 ± 0.06^b^	˂0.001
Celtra Duo		1.67 ± 0.07^B^	1.34 ± 0.07^b^	˂0.001
Katana Zirconia		1.09 ± 0.03^B^	0.83 ± 0.02^b^	˂0.001

Groups with same uppercase letter in the same column indicate significant difference (*P*˂0.05). Groups with same lowercase letter in the same column indicate significant difference (*P*< .05).

The mean difference in translucency parameter (∆TP_00_) after cementation and after coffee staining is shown in Table 5. All groups showed a clinically acceptable difference in translucency parameter (ΔTP_00_ < 2.62) after soaking in coffee solution. In addition, both the *IPS e.max CAD* and *Katana zirconia* groups showed ∆TP_00_ values below the perceptible threshold (ΔTP_00_ ≤ 0.62). The two-way ANOVA test determined a non-significant effect (*P* = 0.151) on ΔTP_00_.

**Table 5:** Mean difference in translucency parameter (∆TP_00_) ± standard deviation of tested groups after cementation and after soaking in coffee solution.

Material	Variolink Esthetic	Relay X Veneer	*P*
IPS e.max CAD	0.62 ± 0.05^A^	0.58 ± 0.13^a^	0.356
Celtra Duo	0.73 ± 0.15^A^	0.68 ± 0.18^a^	0.565
Katana Zirconia	0.46 ± 0.11^A^	0.28 ± 0.04^a^	< 0.001

Groups with same uppercase letter in the same column indicate significant difference (*P*˂0.05). Groups with same lowercase letter in the same column indicate significant difference (*P*˂0.05).

The mean contrast ratio values and standard deviations of all groups are presented in Table 6. The two-way ANOVA test showed that there was no interaction between the ceramic material and the cement type after cementation (*P* = 0.127), however, there was a significant interaction (*P* = 0.001) after soaking in coffee.

**Table 6:** Mean contrast ratio (CR) ± standard deviation of tested groups after cementation and after soaking in coffee solution.

Group	After cementation	After Coffee
EV	0.95 ± 0.04	0.97 ± 0.03
ER	0.96 ± 0.03	0.97 ± 0.02
CV	0.96 ± 0.03^A^	0.97 ± 0.03
CR	0.97 ± 0.02^A^	0.98 ± 0.02
KV	0.98 ± 0.03^B^	0.98 ± 0.03
KR	0.98 ± 0.03^B^	0.99 ± 0.02

Groups with same uppercase letter in the same column indicate significant difference (*P*˂0.05).

## Discussion

The results of the present investigation support rejection of the first null hypothesis, as differences in color and translucency were found in ceramic laminate veneers bonded using resin cements that contain different photoinitiators. The second null hypothesis was also rejected as the color stability and translucency of the tested restorations were impaired after soaking in coffee solution.

In the current study, identical resin abutments with uniform facial reduction were used because they allow easy standardization of the color, size, and shape of the abutments^52,53^. Although natural teeth are more representative of clinical conditions during in vitro testing, they are much more difficult to use due to its variations in color, size, and shape, making it difficult to standardize the experiment. Additionally, collecting sound extracted human teeth of comparable size, shape, and color requires significant time and raises ethical concerns^15^. Song et al.^54^ studied the water sorption and color stability of CAD-CAM milled polymethyl methacrylate materials and reported that the water sorption of these materials may have little effect on its color stability. Furthermore, they concluded that the degree of discoloration of these materials, after soaking for eight weeks in coffee and black tea staining solutions, increased with time. In the current study, the specimens were soaking in coffee solution for a short period (18 h). The soaking in coffee solution was selected based on the following estimate: 3 coffees per day times 1 min of exposure per cup times 365 days per year, for a total of 1095 min, or more than 18 h of exposure per year^50^.

The reflectance spectrophotometer was used in the study to detect color and translucency by acquiring the CIE coordinates of the ceramic restoration, as it has been used in dental research with high accuracy and precision in color measurement^12,21,39,43^. The contrast ratio was used to quantify the translucency^32^. Plasticine was used as backing, as the surface of the restoration was not uniform, to control the edge loss effect during the measurement of color and translucency^33^. The CIE DE2000 color difference formula was used for the calculations of color difference and translucency parameter because it has an interaction expression between hue and chroma to improve the performance of blue colors^38^. The CIE DE2000 formula was the recommended color difference measurement formula for clinical applications and evaluation of color difference thresholds and color and translucency for dental ceramics^34,36^.

Two light cure resin cements with translucent shade containing two different photoinitiators (ivocerindibenzoyl germanium and diphenyliodonium hexafluorophosphate with ethyl-4-dimethylamino benzoate) were selected for the study^29,30^. Camphorquinone, the most commonly used photoinitiator, requires a co-initiator like tertiary amine, but its main disadvantage is yellowish discoloration^24^. The dual cure resin cement had lower color stability than light cure resin cement associated with the chemical initiator amine, which is prone to degradation and the presence of unreacted camphorquinone resulting in a yellowish discoloration^29,30^. The use of translucent shade resin cement was recommended as it exhibited significantly less color change than other shades^16^. The restorations were subjected to thermocycling and load cycling prior to soaking in the staining solution as a clinically relevant protocol to simulate the oral environment^49,50^. The soaking in coffee solution was used to simulate clinical discoloration from commonly consumed beverages with high pigmentation potential^37,42,50^.

The results of the current study showed that L* values decreased while a* and b* values increased for all groups after soaking in coffee solution indicating that the restorations become darker, more reddish, and more yellowish. In addition, TP_00_ values decreased and CR increased for all groups. These findings were supported by the results of previous studies^16,19^. The color changes of all groups were above the acceptability threshold (ΔE_00_ > 2.25). The mean values of the color difference of the lithium disilicate groups were 3.25 ± 0.03 and 3.01 ± 0.07 for *Variolink Esthetic and RelyX Veneer,* respectively. This result is consistent with Tuncdemir et al.^15^ who reported a mean color change of 3.31 ± 2.56 after aging for lithium disilicate ceramic laminate veneers with minimal tooth preparation. In the present study, all specimens were subjected to artificial aging prior to soaking in coffee solution. The discoloration caused by the accelerated artificial aging methods has been linked to the hydrolytic degradation of organic components in resin-based materials, mostly with a chemical degradation of the polymerization promoters^21,26^. Additionally, the presence of unreacted or oxidized molecules from the polymerization system can influence the optical properties of resin-based materials^25^.

The color difference was influenced by material type, with the least color changes associated with lithium disilicate groups, followed by *Celtra Duo* and *Katana Zirconia* groups. According to a previous study, *IPS e.max CAD* has less color changes than *Celtra Duo*^20^. It was found that the color change of *IPS e.max CAD* was less than that of *Celtra Duo* and translucent zirconia^37^. A previous study showed that lithium disilicate revealed a smoother surface and higher color stability after glazing or polishing compared to other chair-side materials^51^. In addition, the highly translucent zirconia was less color stable than *IPS e.max CAD* in distilled water and various mouthwashes^18^. The effect of mouthwashes on the color of different ceramics in two thicknesses was investigated and it was found that translucent zirconia has lower color stability than *IPS e.max CAD* in the thickness of the laminate veneer^3^.

The translucency of ceramic laminate veneers was influenced by the material type after cementation and after staining, and the changes in translucency were clinically acceptable (ΔTP_00_ > 2.62). *IPS e.max CAD* veneer was the most translucent material with the smallest drop color coordinates. It has a unique microstructure that contain a large amount of a glassy phase and a relatively translucent crystal that could interfere with the polymerization of the resin cements^40^. The present study revealed that there was no significant difference in translucency between *IPS e.max CAD* and *Celtra Duo* after cementation, however there was a significant difference after staining. This finding is supported by previous studies^11,17,19,37^. The results of the present study also showed a significant difference in translucency between *IPS e.max CAD* and *Katana zirconia UTML* after cementation and after aging. This finding is supported by previous studies^9,10,14^. The least observed change in translucency was found in *Katana zirconia*. Additionally, cubic zirconia has been reported to be a stable phase, resistant to hydrothermal aging^7,8^.

Both resin cements in this study contain TEGDMA and Bis-GMA monomers with chemical structure susceptible to hydrolysis and/or hydrogen bridging with water^21^. Hydrolytic degradation and hygroscopic effects are determinants of color changes in resin-based materials^18^. The results of the present study showed that the color difference and translucency of ceramic laminate veneers were significantly influenced by the type of resin cement. The *Variolink Esthetic* groups had higher ΔE_00_ values and higher TP_00_ values after cementation and after soaking in coffee solution compared to the *RelyX Veneer* groups. This result is supported by Espíndola-Castro et al.^27^ who reported higher color difference values for *Variolink Esthetic* than for *RelyX Veneer* after soaking in coffee solution. However, the *Variolink Esthetic* cement contains a different photoinitiator (*Ivocerin*) that reacts with monomers to promote polymerization when exposed to light, resulting in increased polymerization, improved reactivity to the curing light, and greater depth of cure^35^. A previous study reported that *Variolink Esthetic* cement shows different color changes depending on the media with the greatest color change after soaking in coffee solution^28^. In terms of opacity, CR increased after staining while translucency decreased. It was reported that the CR value is negatively correlated with the TP_00_ value^33^. The *RelyX Veneer* groups showed high CR values, which was expected since the manufacturer classifies this material as opaque. The increased opacity after aging is consistent with other studies^19,31^.

The limitations of the current study are that it was an in vitro study and the CAD-CAM milled polymethyl methacrylate abutments were used without coating. The surfaces of the abutments uncovered by the restoration were subjected to staining. It is to be noted that resin abutments behave differently in term of their optical behavior and stainability. Thus, different changes in color and translucency can be predicted when natural teeth are used. Furthermore, two types of resin cements were used with a limited aging time. Future clinical studies are needed to evaluate the color and translucency changes of different ceramic laminate veneers and different types of resin cements with different shades.

## Conclusions

Within the limitations of the current study, the following conclusions can be obtained:

1. The resin cement affects the color and translucency of ceramic laminate veneers used for diastema closure, and ceramic laminate veneers bonded with *Variolink Esthetic LC* resin cement are more translucent, while ceramic laminate veneers bonded with *RelyX Veneer* resin are more resistant to coffee staining.

2. The lithium disilicate laminate veneer is more resistant to coffee staining than zirconia reinforced lithium silicate and translucent zirconia laminate veneers used for diastema closure.

## Data Availability

The datasets used and/or analysed during the current study are available from the corresponding author on reasonable request.
